# Monthly variation in the probability of presence of adult *Culicoides* populations in nine European countries and the implications for targeted surveillance

**DOI:** 10.1186/s13071-018-3182-0

**Published:** 2018-11-29

**Authors:** Ana Carolina Cuéllar, Lene Jung Kjær, Andreas Baum, Anders Stockmarr, Henrik Skovgard, Søren Achim Nielsen, Mats Gunnar Andersson, Anders Lindström, Jan Chirico, Renke Lühken, Sonja Steinke, Ellen Kiel, Jörn Gethmann, Franz J. Conraths, Magdalena Larska, Marcin Smreczak, Anna Orłowska, Inger Hamnes, Ståle Sviland, Petter Hopp, Katharina Brugger, Franz Rubel, Thomas Balenghien, Claire Garros, Ignace Rakotoarivony, Xavier Allène, Jonathan Lhoir, David Chavernac, Jean-Claude Delécolle, Bruno Mathieu, Delphine Delécolle, Marie-Laure Setier-Rio, Roger Venail, Bethsabée Scheid, Miguel Ángel Miranda Chueca, Carlos Barceló, Javier Lucientes, Rosa Estrada, Alexander Mathis, Wesley Tack, René Bødker

**Affiliations:** 1 Division for Diagnostics and Scientific Advice, National Veterinary Institute, Technical University of Denmark (DTU), Lyngby, Denmark; 2 Department of Applied Mathematics and Computer Science, Technical University of Denmark (DTU), Lyngby, Denmark; 3 Department of Agroecology - Entomology and Plant Pathology, Aarhus University, Aarhus, Denmark; 4 Department of Science and Environment, Roskilde University, Roskilde, Denmark; 5 National Veterinary Institute (SVA), Uppsala, Sweden; 6 Bernhard Nocht Institute for Tropical Medicine, WHO Collaborating Centre for Arbovirus and Hemorrhagic Fever Reference and Research National Reference Centre for Tropical Infectious Diseases, Hamburg, Germany; 7 Department of Biology and Environmental Sciences, Carl von Ossietzky University, Oldenburg, Germany; 8 Institute of Epidemiology, Friedrich Loeffler Institute, Greifswald, Germany; 9 Department of Virology, National Veterinary Research Institute, Pulawy, Poland; 10 Norwegian Veterinary Institute, Oslo, Norway; 11 Institute for Veterinary Public Health, Vetmeduni, Vienna, Austria; 12 CIRAD, UMR ASTRE, F-34398 Montpellier, France; 13 Institute of Parasitology and Tropical Pathology of Strasbourg, EA7292, Université de Strasbourg, Strasbourg, France; 14 EID Méditerranée, Montpellier, France; 15 Laboratory of Zoology, University of the Balearic Islands, Palma, Spain; 16 Department of Animal Pathology, University of Zaragoza, Zaragoza, Spain; 17 Institute of Parasitology, University of Zürich, Zürich, Switzerland; 18 Avia-GIS NV, Zoersel, Belgium

**Keywords:** *Culicoides*, Random Forest, Machine Learning, Europe, Monthly distribution, Spatial distribution, Presence-absence data, Targeted surveillance

## Abstract

**Background:**

Biting midges of the genus *Culicoides* (Diptera: Ceratopogonidae) are small hematophagous insects responsible for the transmission of bluetongue virus, Schmallenberg virus and African horse sickness virus to wild and domestic ruminants and equids. Outbreaks of these viruses have caused economic damage within the European Union. The spatio-temporal distribution of biting midges is a key factor in identifying areas with the potential for disease spread. The aim of this study was to identify and map areas of neglectable adult activity for each month in an average year. Average monthly risk maps can be used as a tool when allocating resources for surveillance and control programs within Europe.

**Methods:**

We modelled the occurrence of *C. imicola* and the Obsoletus and Pulicaris ensembles using existing entomological surveillance data from Spain, France, Germany, Switzerland, Austria, Denmark, Sweden, Norway and Poland. The monthly probability of each vector species and ensembles being present in Europe based on climatic and environmental input variables was estimated with the machine learning technique Random Forest. Subsequently, the monthly probability was classified into three classes: Absence, Presence and Uncertain status. These three classes are useful for mapping areas of no risk, areas of high-risk targeted for animal movement restrictions, and areas with an uncertain status that need active entomological surveillance to determine whether or not vectors are present.

**Results:**

The distribution of *Culicoides* species ensembles were in agreement with their previously reported distribution in Europe. The Random Forest models were very accurate in predicting the probability of presence for *C. imicola* (mean AUC = 0.95), less accurate for the Obsoletus ensemble (mean AUC = 0.84), while the lowest accuracy was found for the Pulicaris ensemble (mean AUC = 0.71). The most important environmental variables in the models were related to temperature and precipitation for all three groups.

**Conclusions:**

The duration periods with low or null adult activity can be derived from the associated monthly distribution maps, and it was also possible to identify and map areas with uncertain predictions. In the absence of ongoing vector surveillance, these maps can be used by veterinary authorities to classify areas as likely vector-free or as likely risk areas from southern Spain to northern Sweden with acceptable precision. The maps can also focus costly entomological surveillance to seasons and areas where the predictions and vector-free status remain uncertain.

**Electronic supplementary material:**

The online version of this article (10.1186/s13071-018-3182-0) contains supplementary material, which is available to authorized users.

## Background

*Culicoides* (Diptera: Ceratopogonidae) biting midges are small blood-sucking insects responsible for the transmission of viruses causing the European outbreaks of bluetongue (BT) and Schmallenberg diseases in wild and domestic ruminant livestock [1, 2], and for African horse sickness in equids [1, 3]. BTV historically made sporadic incursions into some countries of the Mediterranean Basin (Portugal, Spain, the Greek islands close to Turkey and Cyprus) but from 1998 onwards the situation worsened when five other serotypes spread within France (Corsica), Italy, Greece and countries in the Balkans region [4]. BT was never reported in northern Europe until August 2006, when an unprecedented bluetongue virus (BTV) serotype 8 outbreak started in the border region of Germany, Belgium and the Netherlands and, over the next two years, it spread further over central and northern Europe [5–8]. This epidemic had a significant economic impact within the European Union, as a consequence of the restriction of animal movements and the large amount of financial resources invested in vaccination campaigns and vector surveillance programs [9–11]. In northern Europe, the Afro-Asian vector *Culicoides imicola* Kieffer is absent and therefore, the vector species incriminated in the transmission of BTV were the Palaearctic species belonging to the Obsoletus ensemble *Culicoides obsoletus* (Meigen)/*Culicoides scoticus* Downes & Kettle [12, 13], *Culicoides chiopterus* (Meigen) [14, 15] and *Culicoides dewulfi* Goetghebuer [16].

Many factors contribute to the transmission of vector-borne diseases, including the presence of infected hosts, competent vectors and suitable environmental temperatures for the pathogen to replicate inside the vector [17]. In the absence of ongoing entomological surveillance, a temporal map of the potential distribution of the vectors is key for health authorities to quickly delimitate possible areas and time periods of risk for disease transmission in the case of an outbreak of a known or emerging vector-borne disease [18–20]. The spatial distribution and phenology of vectors can be predicted from climate and environmental variables such as temperature, precipitation and land cover [18]. Temporal occurrence data (the presence or absence of a species at a specific time) in non-sampled areas or periods can be modelled using statistical techniques. This methodology is used to generate species distribution maps depicting the probability of the species being present at a given time [21], thus identifying areas with low or null adult activity and therefore, periods during which animal movements are safe.

Since the start of the BT outbreaks, European authorities have established a series of regulations for BT surveillance including vector monitoring to analyse the seasonal fluctuation of the vector populations and determine the seasonal vector-free periods (SVFP) for different regions [22, 23]. The EU defines SVFP by using a threshold on the abundance of female specimens, considering the parity stage of the *Culicoides* caught in the traps. This approach has been used to estimate the SVPF in Scotland for species of the Obsoletus group [24]. The authors estimated phenological events for each species such as the start and end of the SVFP. Brugger et al. [23] estimated vector-free periods in Austria using an approach based on the European Commission definition but without considering parity stage of female specimens. In the present study, we identified months where adult activity is null or very low, based on the monthly mean abundance for each farm, without considering the parity of the specimens collected as previously proposed by the EU legislation. Our definition of adult activity is different but comparable to the vector-free season defined by this legislation and, therefore, we keep the term “vector-free” season or period to refer to a period of the year with neglectable adult activity.

The SVFP during the winter was not ubiquitous across all European countries. Austria [23], Switzerland [25] and Sweden [26] reported the existence of a SVFP, while other countries such as Germany, France, Belgium and the Netherlands reported that a SVFP might not exist in these countries [16, 27–29]. Imposing restrictions of animal movement in areas where the vector is not present has a negative economic impact as the restriction is unnecessary. On the other hand, allowing animal movement in areas where the vector is present poses a risk of spreading infections to new areas, if environmental conditions are suitable for the virus to develop inside the vector. Being able to define vector-free areas and periods is not only useful for BT management, but also for emerging *Culicoides*-borne diseases in the future. For instance, Schmallenberg virus appeared suddenly in 2011 in Germany, and spread throughout 29 European countries [30], causing economic losses for sheep and cattle farmers [31]. In addition, the spread of African horse sickness has previously been reported in horses in Spain in 1966 and Spain and Portugal from 1987 to 1990 [32]. Knowing the geographical distribution of vectors allows veterinary authorities to focus control measurements in those areas at a specific time of year.

In this study, we used entomological data of *C. imicola*, Obsoletus ensemble and Pulicaris ensemble collected from nine European countries over a seven-year period. This entomological dataset was used previously to analyse the temporal fluctuation at different latitude bands for Europe, to analyse the start of the season at the geographical NUTS level and to interpolate the observed *Culicoides* abundance spatially [32]. In this work, we use the machine learning algorithm “Random Forest” (RF) to model the average monthly presence/absence observed and predict the probability of presence of *C. imicola*, Obsoletus ensemble and Pulicaris ensemble in unsampled areas, using climatic and environmental variables as predictors. The aim of this work was to predict areas and months likely to be free of biting midges or likely to have vectors as well as areas of uncertain status that need to be targeted for entomological surveillance in case of an outbreak. The resulting maps represent the first spatial distribution model for a transect comprising nine European countries from southern Spain to northern Sweden. The maps are useful tools as inputs for decision making by veterinary authorities to detect areas with adult activity and use this information to focus financial resources for active entomological surveillance programs.

## Methods

### *Culicoides* data

We used entomological data collected in farms from Spain, France, Germany, Switzerland, Austria, Denmark, Sweden, Norway and Poland between 2007 and 2013 as part of national surveillance programs or research projects [33]. For each trap site, observations consisted of the number of *C. imicola*, Obsoletus ensemble [*C. obsoletus*, *C. scoticus*, *Culicoides montanus* Shakirzjanova, *Culicoides chiopterus* (Meigen) and *C. dewulfi*] and Pulicaris ensemble [*Culicoides pulicaris* (Linnaeus) and *Culicoides punctatus* (Meigen)]. *Culicoides* biting midges were sampled from a total of 904 livestock farms comprising 31,429 trap collections. Onderstepoort traps were used for sampling biting midges, except for Germany (Biogents Sentinel traps) and in Spain (mini CDC traps). For these two countries, we multiplied the number of *Culicoides* for each observation by a conversion factor to make the number of specimens comparable between the different trapping methods. Details of both the sample protocols and the conversion factors used have been published previously [33].

For *C. imicola* and each of the *Culicoides* ensembles, we split the observation data set into 12 subsets according to month of the year. For each 12 monthly dataset, we calculated the average abundance on each farm for each year sampled. This resulted in 12 datasets with farms containing one monthly average abundance per year sampled. Then, we classified each monthly average each year into Presence or Absence according to the average abundance of the vector. Based on the European Union regulation [22] for the definition of the SVFP, in which an abundance threshold of biting midges is proposed to define Presence or Absence, we considered each monthly average for each year as Presence when it was above or equal to an abundance threshold of five midges for the Obsoletus and Pulicaris ensembles, and one specimen for *C. imicola*. Even though the European Union definition of Presence is based on the catch of five parous specimens per observation, we here considered the number of midges without differentiating females into their gonotrophic stage because this information was missing for some of the countries. This will result in a more conservative definition of SVFP. Our approach also differed from the approach used by the EU commission as for each farm we only classified the monthly average each year into Presence or Absence, and not each of the individual observations (when there were several observations per month).

**Fig. 1:**
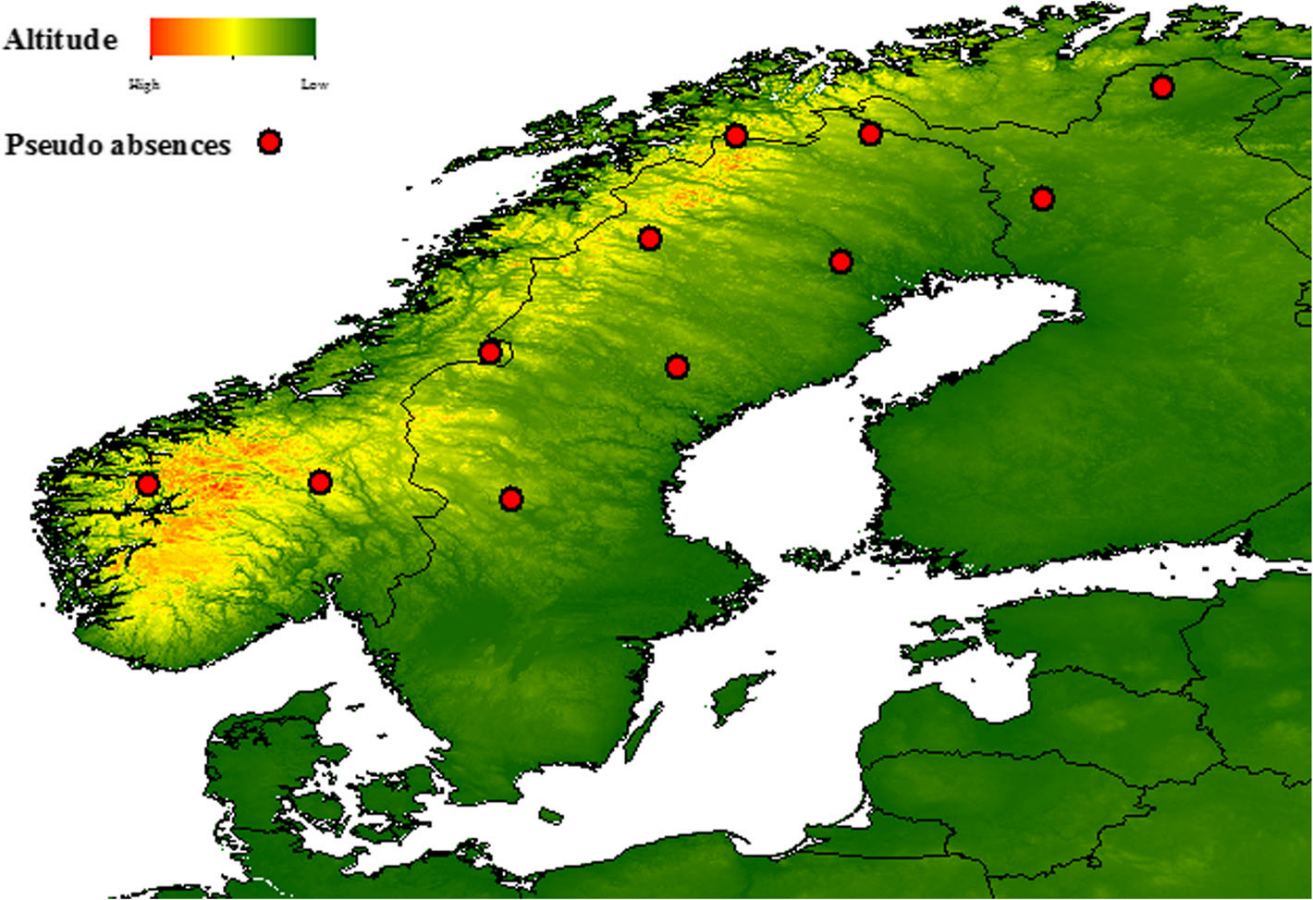
Eleven pseudo-absence points added to Norway, Sweden and Finland for January and February

We constructed preliminary Random Forest (RF) models using occurrence data from January and February. The data collected in this period did not include any farms from northern Scandinavia. The resulting models predicted the occurrence of biting midges in January and February in this region (data not shown). However, earlier studies have reported an absence of biting midges in the Scandinavian peninsula during winter [26, 34]. Therefore, it was useful to provide pseudo-absence points to the models in order to increase their accuracy for predicting absences in the area. For January and February, we created 11 random pseudo-absence points above 60 degrees latitude in the highlands in Norway, central and northern Sweden and Finland and were added by hand using ArcMap 10.1 (ESRI, Redlands, CA, USA) (Fig. 1).

### Predictor variables

We used raster files (images) of 112 environmental and climatic variables, land cover and livestock density, each with a 1 km^2^ spatial resolution.

The environmental predictors included Mid-infrared (MIR), daytime Land Surface Temperature (dLST), nighttime Land Surface Temperature (nLST), Enhanced Vegetation Index (EVI) and Normalized Difference Vegetation Index (NDVI) as predictor variables. Each variable was derived from a MODIS temporal series from 2001 to 2012, and subjected to Temporal Fourier Analysis (FTA) [35]. For each environmental variable, the resulting products of FTA were the 14 images described in Table 1. This dataset was originally created by the TALA research group at the Department of Zoology at Oxford University, and was provided through the EDENext project [36].

**Table 1:** Products of Temporal Fourier Analysis obtained from a single variable

Fourier component	Description
A0	Fourier mean for the entire time series
A1	Amplitude of annual cycle
A2	Amplitude of bi-annual cycle
A3	Amplitude of tri-annual cycle
P1	Phase of annual cycle
P2	Phase of bi-annual cycle
P3	Phase of tri-annual cycle
DA	Proportion of total variance due to all three cycles
D1	Proportion of total variance due to annual cycle
D2	Proportion of total variance due to bi-annual cycle
D3	Proportion of total variance due to tri-annual cycle
MN	Minimum value
MX	Maximum value
VR	Total variance

Each product corresponds to a raster image (1 km^2^ resolution) derived from a single environmental variable (for instance, NDVI)

We also included WORLDCLIM altitude data (digital elevation model) and bioclimatic variables as climatic predictors for *Culicoides* distribution. BIOCLIM images were obtained from the WORLDCLIM database [37, 38] (Table 2).

**Table 2:** MODIS Fourier-transformed, BIOCLIM and Corine Land Cover predictors used to model the probability of *Culicoides* presence

Source	Code	Description
MODIS (Fourier transformed) 2001–2012	MIR	Mid-infrared
	dLST	Daytime land surface temperature
	nLST	Nighttime land surface temperature
	NDVI	Normalized difference vegetation index
	EVI	Enhanced vegetation index
BIOCLIM 1960–1990	BIO 1	Annual mean temperature
	BIO 2	Mean diurnal range: mean of monthly (max. temp - min. temp)
	BIO 3	Isothermality (BIO2/BIO7) (×100)
	BIO 4	Temperature seasonality (standard deviation × 100)
	BIO 5	Maximum temperature of warmest month
	BIO 6	Minimum temperature of coldest month
	BIO 7	Temperature annual range (BIO5-BIO6)
	BIO 8	Mean temperature of wettest quarter
	BIO 9	Mean temperature of driest quarter
	BIO 10	Mean temperature of warmest quarter
	BIO 11	Mean temperature of coldest quarter
	BIO 12	Annual precipitation
	BIO 13	Precipitation of wettest month
	BIO 14	Precipitation of driest month
	BIO 15	Precipitation seasonality (coefficient of variation)
	BIO 16	Precipitation of wettest quarter
	BIO 17	Precipitation of driest quarter
	BIO 18	Precipitation of warmest quarter
	BIO 19	Precipitation of coldest quarter
	Altitude	Digital elevation model (DEM)
Corine Land Cover^a^	CLC 12	Non-irrigated arable land
	CLC 13	Permanently irrigated land
	CLC 15–17	Vineyards, fruit trees and berry plantations, olive groves
	CLC 18	Pastures
	CLC 19	Annual crops associated with permanent crops
	CLC 20	Complex cultivation patterns
	CLC 21	Land principally occupied by agriculture with significant areas of natural vegetation
	CLC 22	Agro-forestry areas
	CLC 23	Broad-leaved forest
	CLC 24	Coniferous forest
	CLC 25	Mixed forest
	CLC 26	Natural grasslands
	CLC 29	Transitional woodland-shrub
	CLC 35	Inland marshes
	CLC 40	Water courses
	CLC 41	Water bodies

^a^CLC plus the number refers to the CORINE land cover class used for modelling

We used a Corine Land Cover (CLC) map with 250 m pixel resolution to extract information on 16 relevant land cover classes (Table 2). For each class, we created a binary image with pixel values of 1 and 0 according the presence or absence of the class. Due to the higher spatial resolution of the CLC map compared to the other predictors, we resampled each of the binary class images to a resolution of 1 km^2^. This was done by overlaying a grid with cells of 1 km^2^ resolution. To each of these cells, we assigned the sum of all pixels with a value of 1 within them. Each 1 km^2^ cell of the grid was made up of 16 (4 × 4) pixels of the original CLC map. This resulted in new images for each land cover class with a pixel resolution of 1 km^2^, representing the frequency of each of the 16 different classes found in every 1 km^2^ area (pixel) on a scale of 0–16. CLC map was obtained from the European Environment Agency website [39].

We obtained livestock density data for cattle, goats, sheep, small ruminants and chickens from the Food and Agriculture Organization repository “GeoNetwork”. This dataset consisted of a series of raster files with information regarding livestock density at a global scale (“The gridded livestock of the world”) [40].

### Modelling the probability of presence

Combining our *Culicoides* data with the predictors, we explored modelling approaches using VECMAP© software, v.2.0.16350.2473. For the final modelling of each month and each species, we used the Random Forest (RF) machine learning technique [41, 42] in R v.3.4.2 [43] (packages *caret* [44] and *randomForest* [45]) to model the probability of presence (PP) in the nine European countries using the Presence/Absence observations calculated at each farm. For each month we obtained a map showing the PP at the same resolution as the predictors (1 km^2^). The RF algorithm consists of an ensemble of decision trees used to predict the probability of class membership where the response variable is categorical (e.g. classification into presence and absence). An advantage of RF is the model’s capability of detecting nonlinear relationships between the response and the predictor variables [46] and that RF can handle a large number of predictor variables [46]. In addition, RF can produce a list of the most important predictors and scale them from 0–100 according to their importance as calculated by permuting each predictor and measuring the prediction error after the permutation [44].

The number of farms sampled varied from month to month. As expected, during summer more farms were sampled compared to winter, as in many countries of northern Europe entomological surveillance is not carried out during the cold winter months. For each monthly dataset, we used a stratified random split to divide the data into two subsets: one included 70% of the farms containing at least one year classified as presence together with the farms with only absence observations (training set). The second subset contained the remaining 30% of the farms as a test set to evaluate model performance [42, 47, 48]. We conducted a stratified random split based on farm ID in order to avoid having observations belonging to the same farm in both the training and the evaluation datasets (Table 3).

**Table 3:** Total number of farms sampled each month and number of farms in the training and test sets

Month	Total no. of sampled farms	Training set (70%)	Test set (30%)
January	444	310	134
February	457	319	138
March	473	331	142
April	522	364	158
May	527	368	159
June	518	362	156
July	581	406	175
August	636	445	191
September	620	433	187
October	522	365	157
November	500	349	151
December	448	313	135

All observations belonging to a single farm were included in either the training or test set, but never in both

The number of *Culicoides* caught per farm highly varied between the different years. In this work, we considered each farm’s monthly classification into Presence or Absence for each year and included them in the training set as independent observations. Therefore, a farm might contain Presence and Absence observations from different years depending on the variation in mean monthly abundance between the different years.

The monthly Presence/Absence data were highly imbalanced, meaning that it contained a high proportion of one of the classes (Presence or Absence), i.e. the majority class. We investigated and compared five different balancing methods (no balancing, down-sampling, oversampling, ROSE [49], SMOTE [50], Tomek [50]) to cope with the imbalance and to improve model performance. We ran cross-validation (CV) for each balancing method 10 times with different random seeds and the best method was chosen according to highest AUC (data not shown). The balancing method chosen to balance the training set was oversampling, which entails duplicating the observations for the minority class in order to reach the same number of observations as the majority class [42]. We used the balanced training set of each month to train the RF model, and used the test sets to calculate the receiver operating characteristics (ROC) curve [42, 51, 52] and the area under this curve (AUC). We used the AUC as a measurement of model performance. AUC values close to 0.5 indicate that the model is not able to classify new samples better than random, values between 0.7 and 0.8 indicate acceptable model performance, values from 0.8 to 0.9 indicate excellent performance and values above 0.9 are considered outstanding [53]. For each month, we performed 5-fold CV to optimize the model parameter “mtry” (i.e. number of predictors used at each split). The “ntrees” parameter (number of trees of the forest) was set to 1000 trees in all cases.

For *C. imicola*, after the test set was created, we removed all the observations from farms not belonging to Spain or France, as the vector was not found in the seven remaining countries [33]. This reduced the large amount of Absence observations in the test set, which have an influence in the distribution of the classes.

### Classification

Classification of predicted probabilities into Presence/Absence classes can be determined using a predetermined threshold (in ecology studies, normally the default is a PP of 0.5 [54]). Here, we were interested in defining a data-dependent threshold, as a predefined threshold of 0.5 might not be optimal [54]. The monthly PP maps obtained from our RF models were classified into three categories. We calculated a lower and upper threshold and all areas with a PP below the lower threshold were considered to be in the Absence class, while the areas with a PP above the upper threshold were classified as Presence areas. Regions with a PP between the two thresholds could not be classified as either Absence or Presence class, and were therefore classified as an Uncertain status category that may be targeted for active vector surveillance. The Absence and Presence classes refer here to the occurrence of adult activity and not to the ecological establishment of the vector, as in the classical species distribution modelling.

Lower and upper thresholds were calculated using the density function for the PP predicted by the model for each test set class (true presence/absence). To define the two thresholds for each month, we derived two gain functions *G*_*presence*_, *G*_*absence*_ for 100 possible thresholds from 0 to 1, based on the area under the density function for Presence and Absence, respectively. We calculated *G*_*presence*_ as the probability of a true presence and subtracted the probability of a misclassified presence multiplied by a parameter *δ*, which indicates the cost of a misclassified presence relative to a true presence. Similarly, we calculated *G*_*absence*_ as the probability of a corrected classified absence (true absence) and subtracted the probability of misclassified absence multiplied by parameter γ, which indicates the cost of a misclassified absence relative to a true absence. Setting δ = 2, for example, means that the cost of a false positive classification is twice the gain of a true positive classification. The gain value can be considered in terms of timely initiation of countermeasures and a lower probability of an epidemic and trade restrictions, while the loss value would be the cost to the farm and society of incorrectly applied countermeasures. Similarly, for the interpretation of γ, the gain of a true negative classification and the loss from a false negative classification can be likened to being declared free from disease, with the cost to both farmer and society of a subsequent discovery of the disease. Similar considerations can be used to relate δ and γ to each other. If, for example, we set δ = ρ * γ in Eq. 1, the cost of misclassifying a presence is ρ times the cost of misclassifying an absence. We assign δ = 2 * γ in order to assign twice the importance to the Presence misclassifications compared to Absence misclassifications and we set γ = 2 to still give some importance to the Absences misclassifications.

The equations for *G*_*presence*_, *G*_*absence*_ were:

1 $$ Gpresence\ \left(\mathrm{q}\right)={\int}_q^1 Presence\ (x)\  dx-\delta \ast {\int}_q^1 Absence(x)\  dx $$

2 $$ Gabsence\ \left(\mathrm{q}\right)={\int}_0^q Absence\ (x)\  dx-\gamma \ast {\int}_0^q Presence(x)\  dx $$

where q represents the possible threshold value between 0 to 1, and where δ and γ are loss parameters.

To calculate the lower threshold, we used Eq. 1 to find the optimal upper threshold when assuming a loss parameter of δ = 4 by optimizing the gain *G*_*presence*_. Similarly, Eq. 2 was used to find the optimal lower threshold, assuming a loss parameter γ = 2. The upper and lower thresholds depend on the predictive power of the model, being more separated when the overlapping between classes is large. If the model performance is good, the overlapping between classes will be less and the two thresholds will be closer together.

In order to evaluate the sensitivity of the thresholds to the distribution of different test sets, we divided each monthly test set into ten equally sized folds (10 subsets) and calculated the density functions using nine out of the ten folds. This procedure was repeated for all the different folds (10 times), excluding a different fold each time, and plotted the new lower and upper thresholds together in the same graph. We applied this 10-fold cross-validation scheme to compare the threshold calculated with different subsets of the test set versus the thresholds calculated using all the observations of the test set.

We classified the monthly probability maps into the three classes: “Absence”, “Uncertain” and “Presence” using the thresholds calculated from all the observations of the test set.

## Results

### Obsoletus ensemble

**Fig. 2:**
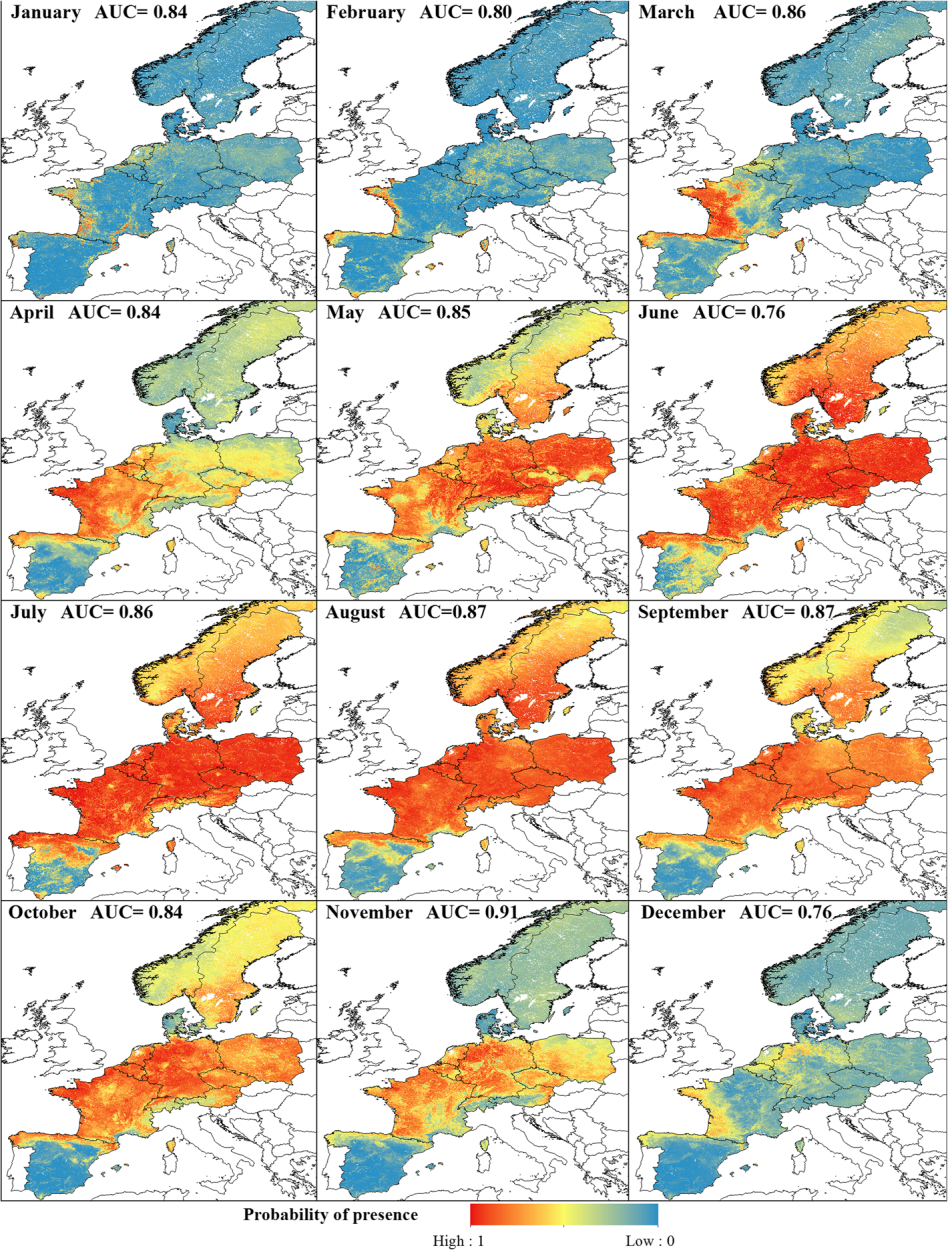
Predicted monthly probability of presence of Obsoletus ensemble. Monthly model performance is shown as the AUC value

The 12 models were shown to perform well for the Obsoletus ensemble, with an AUC ranging from 0.76 in June and December to 0.91 in November (mean AUC = 0.84) (Fig. 2).

**Fig. 3:**
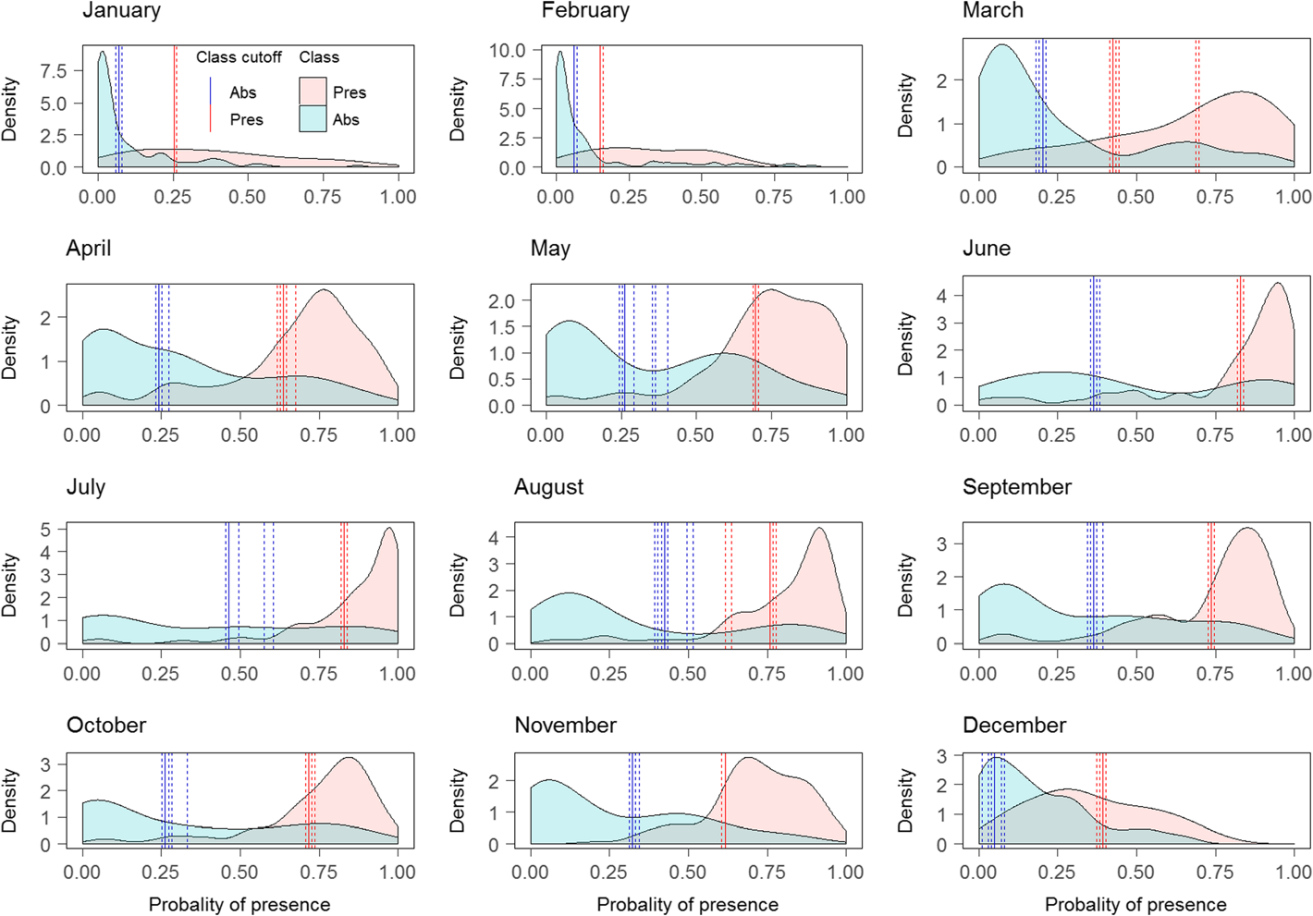
Obsoletus ensemble: monthly distribution of Presence and Absence classes of the test set samples as a function of their predicted probability of presence. Dashed lines show the additional thresholds calculated from 10-fold CV

The majority class shifted from Absence in December-March, to Presence in April-November, and the models generally had good predictive power when predicting the majority class. However, the models performed less well when predicting the minority class. For January and February, the model predicted the Presence class relatively poorly, with a relatively flat density function (Fig. 3). The additional thresholds calculated using 10-fold CV were similar to the main threshold, indicating that the distribution of classes in the test set were robust when subtracting 10% of the data. The lower thresholds showed more variation compared to the variation of the upper thresholds (Fig. 3).

**Fig. 4:**
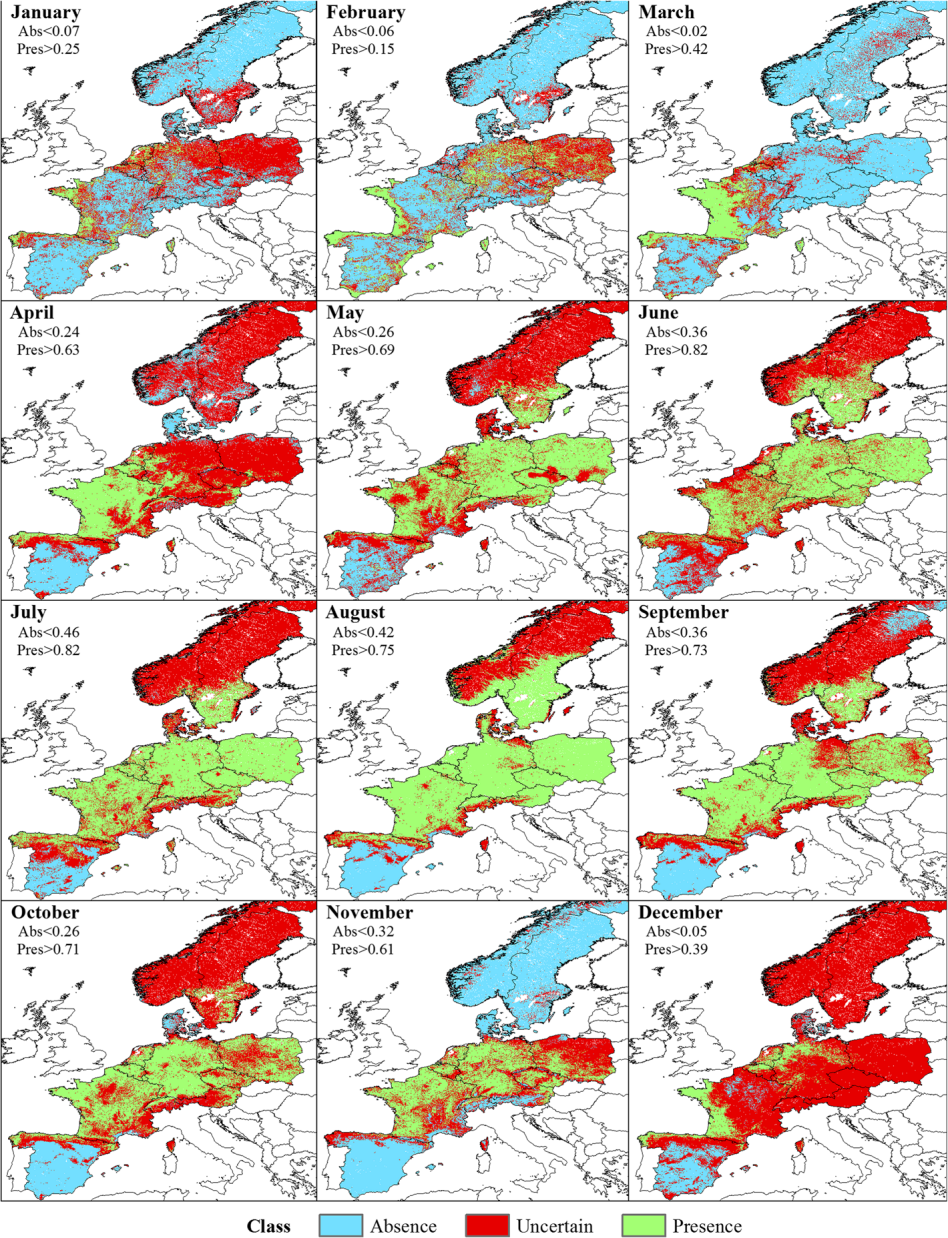
Classification of the predicted probability of presence of Obsoletus ensemble into Absence, Presence and Uncertain areas at a 1 km^2^ resolution

Classifications did not result in clearly delineated geographical zones for the three classes (Presence, Absence and Uncertain), although spatial patterns were observed (Fig. 4). In January, the Obsoletus ensemble was predicted present in areas within the western part of France, northern coast of Spain and in scattered areas of Germany, and it was predicted absent from northern and central Scandinavia, eastern France and parts of Germany. The Uncertain class area was present in southern Scandinavia, eastern Germany and Poland. In February the Presence area in western France and the northern coast of Spain appeared clearly segregated while more dispersed patches appeared in Germany and Poland. The Uncertain class area was reduced to patches in Germany, Poland and a small portion of southern Sweden. During March, the Presence area extended further west into France, while the Absence area was clearly concentrated in the eastern part of Europe and Scandinavia. The Uncertain area was a more coherent intermediate region between these two areas, found in eastern France, Belgium and the Netherlands. In April, the Presence class expanded from western France occupying most of France while the eastern part of the study area and Scandinavia remained in the Uncertain area. From May onwards, the general pattern showed the Obsoletus ensemble to be widely distributed in France, Germany, Austria, Switzerland, Poland and southern Scandinavia. The Absence class areas were located in southern Spain during this period. In November, Scandinavia was classified as an Absence class area together with Spain (except the northern coast of Spain, that was included in Presence area). Finally, in December the Presence class was clustered in western France and some patches in northern Germany while the remaining areas, with exception of southern Spain, appeared classified as Uncertain areas, including the Scandinavian peninsula (Fig. 4).

### Pulicaris ensemble

**Fig. 5:**
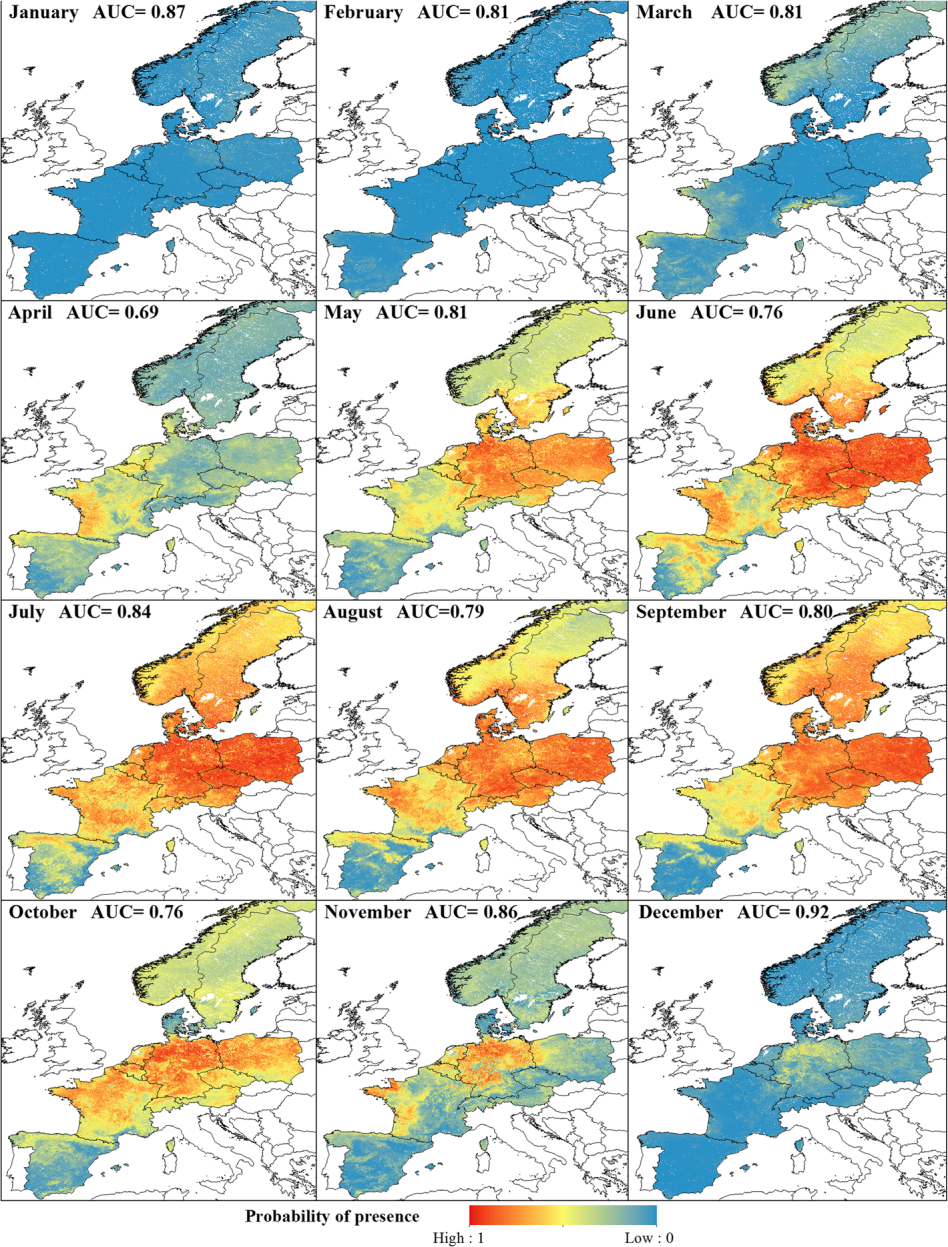
Predicted monthly probability of presence of Pulicaris ensemble. Monthly model performance is shown as the AUC value

The RF models performed less well in predicting the PP for the Pulicaris ensemble. The mean AUC was 0.81, ranging from 0.69 in April to 0.92 in December (Fig. 5).

**Fig. 6:**
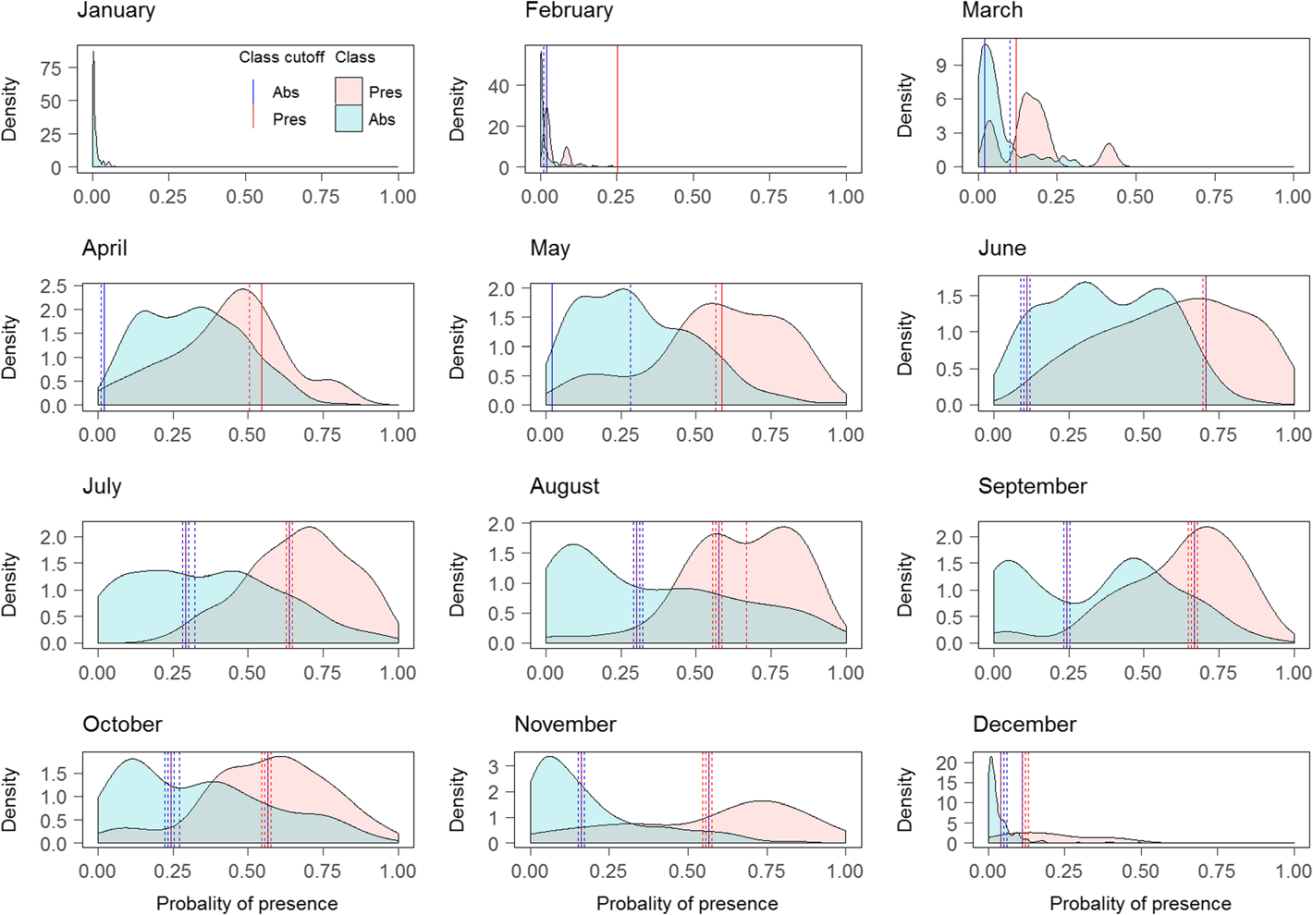
Pulicaris ensemble: monthly distribution of Presence and Absence classes of the test set samples as a function of their predicted probability of presence. Dashed lines show the additional thresholds calculated from 10-fold CV

For January, the test set contained only three Presence observations from a single farm and the density function and thresholds could not be calculated. Therefore, the PP map could not be classified into the three classes. For February, the PP predicted for the observed Presences were completely included within the range of the PP predicted for the Absence class, meaning that the model was incapable of distinguishing the Presence class. Nevertheless, because both density functions were computed, the lower and upper thresholds were still calculated. The distribution of predicted Presence and Absence areas for the Pulicaris ensemble test set contained larger overlapping areas between both distributions than for the Obsoletus ensemble, resulting in poorer predictive power for distinguishing between the classes. For the months of April, May and June, the distribution of both classes overlapped so much that the lower threshold was calculated as close to 0 to avoid false negative classifications (Fig. 6). For the Pulicaris ensemble, the additional thresholds calculated using 10-fold CV, were similar to the main threshold for all the months, meaning that the distribution of classes in the test set were robust when subtracting 10% of the data. Both lower and upper thresholds seemed to be robust for the different test sets (Fig. 6).

**Fig. 7:**
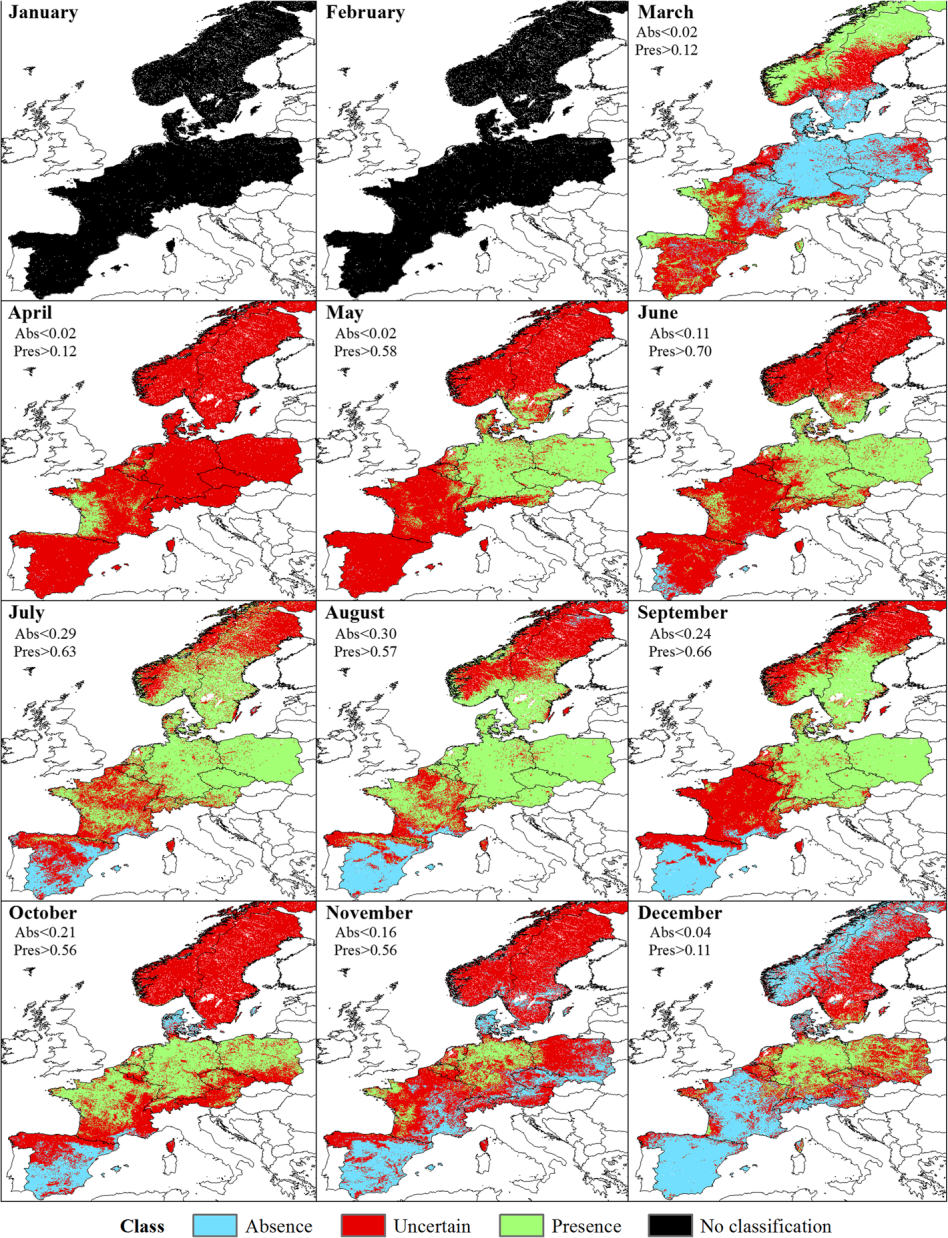
Classification of the predicted probability of presence of Pulicaris ensemble into Absence, Presence and Uncertain areas at a 1 km^2^ resolution

Due to the lack of Presence observations in January, we could not define thresholds for classifying the PP map. In February, because PP of the observed Presence observations were completely included in the range of the PP of the Absence class, we decided not to classify the map as the model was incapable of distinguishing the Presence class and would lead to an incorrect interpretation of the classification. In March, the Pulicaris ensemble was predicted to be present on the west coast of France, northern coast of Spain and in central and northern Scandinavia, while the Absence class was distributed in eastern France, Germany and Poland. The Uncertain area was located between the Presence and Absence class. During April, May and June, the model was able to predict the Presence class but it was incapable of distinguishing the Absence class, resulting in classification only for the Presence and Uncertain class. From July to October, the Presence class extended towards the eastern part of the study area while the Uncertain class occupied northern Scandinavia. During September, the Uncertain class was additionally found in France. In November, the Presence areas were located mostly in Germany and some patches in France while Scandinavia was classified into the Uncertain class. The Absence class was predicted in Denmark and southern Spain. During December, the Absence class was localized in Spain, France and northern Scandinavia while the Presence class remained in some patches in Germany (Fig. 7).

### *Culicoides imicola*

**Fig. 8:**
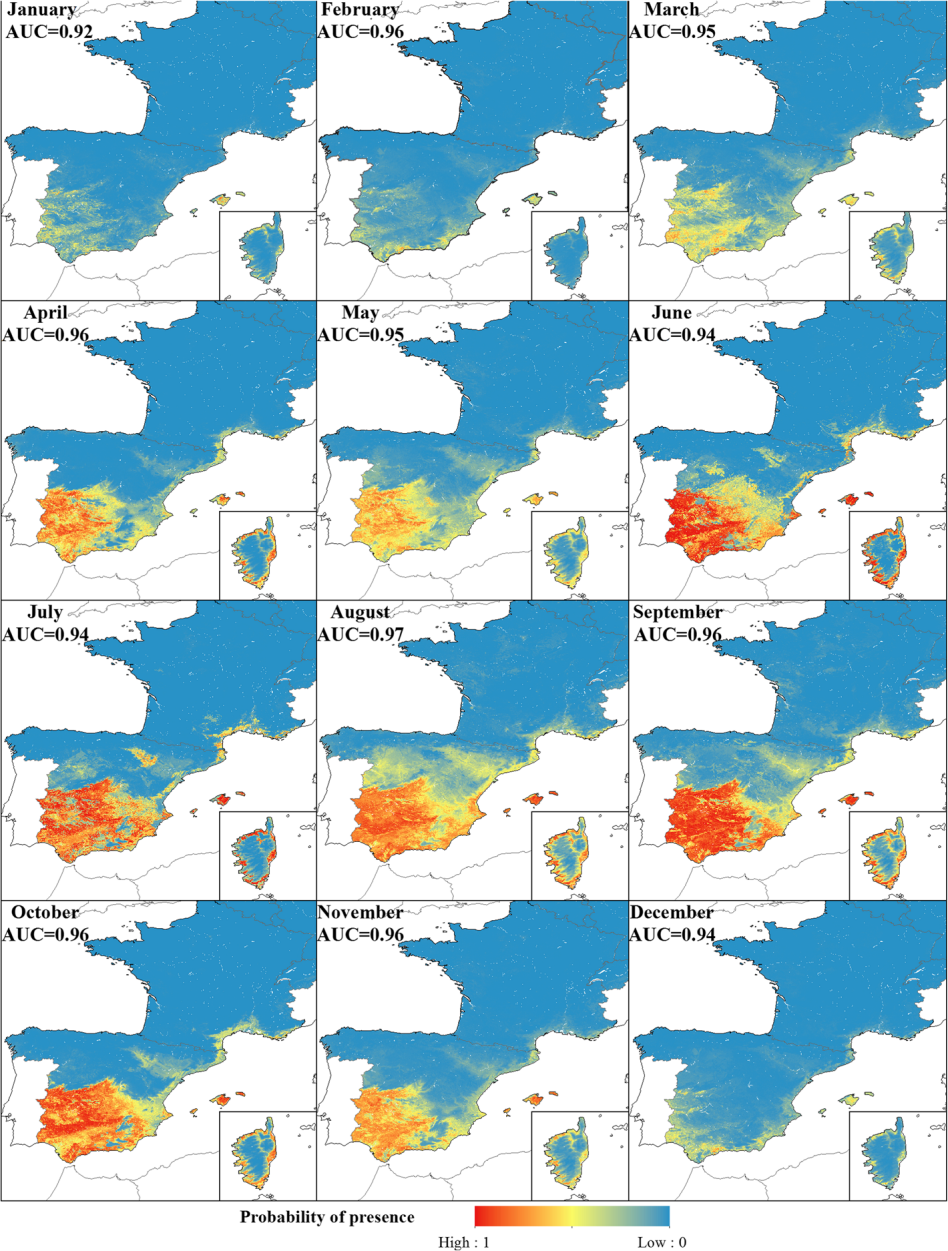
Predicted monthly probability of presence of *C. imicola*. Monthly model performance is shown as the AUC value

The RF models for *C. imicola* had a very high accuracy for distinguishing the Presence and Absence classes. The models had a mean AUC of 0.95, ranging from 0.92 in January to 0.97 in August (Fig. 8).

**Fig. 9:**
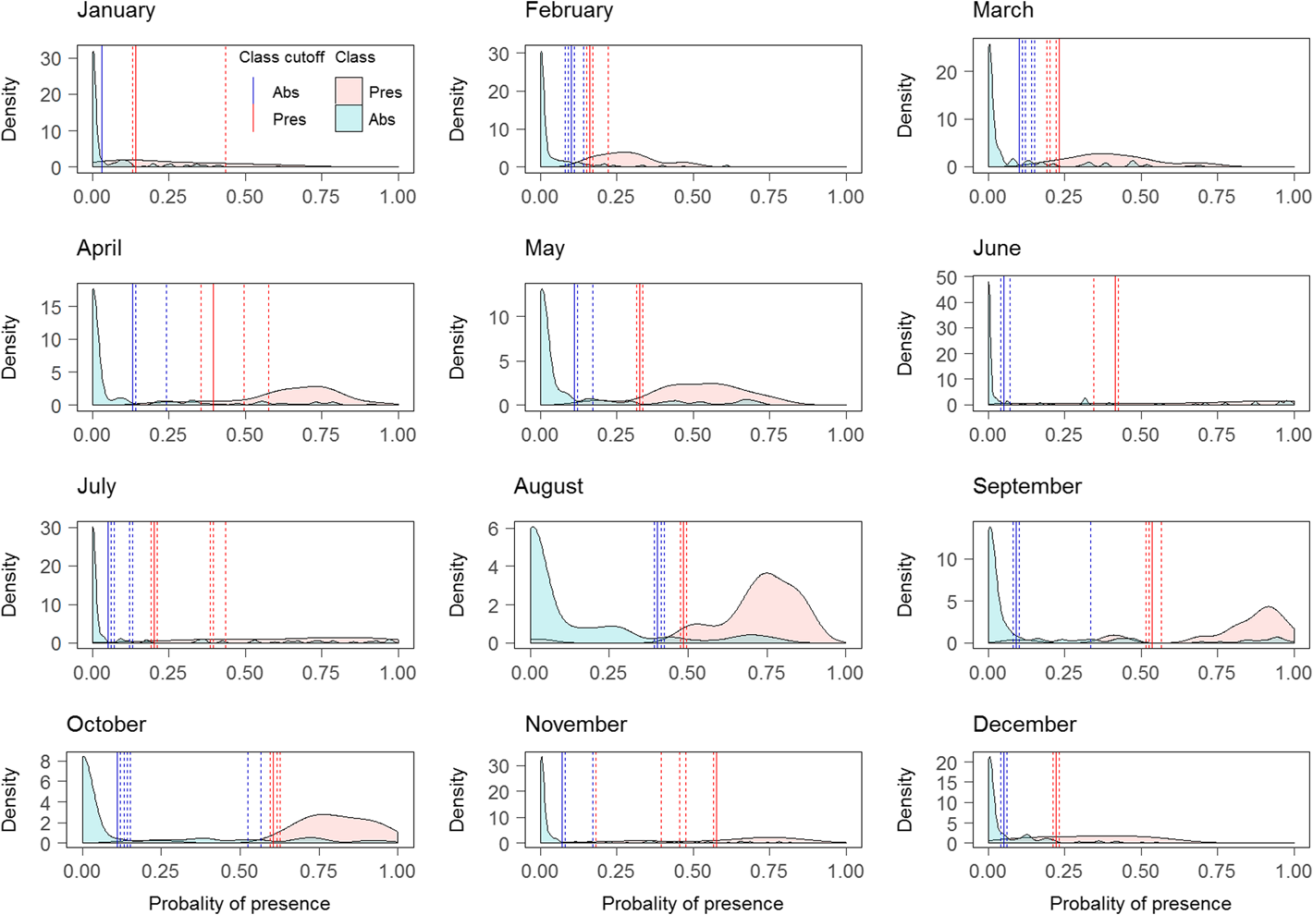
*Culicoides imicola*: monthly distribution of Presence and Absence classes of the test set samples as a function of their predicted probability of presence. Dashed lines show the additional thresholds calculated from 10-fold CV

The RF models predicted the *C. imicola* Absence class very well. Absence constituted the majority class for all months as the species was only found in Spain and southern France. The Presence class was less well predicted, as reflected in a flatter distribution. Nevertheless, the model was able to distinguish both classes, resulting in a narrow area of uncertainty between the lower and upper thresholds (Fig. 9). The additional thresholds calculated using 10-fold CV, were similar to the main threshold, indicating that the distribution of classes in the test set were robust when subtracting 10% of the data. The upper thresholds showed more variation compared to the variation in the lower thresholds. Particularly April, July and November seemed to have upper thresholds sensitive to the class distribution of the test set (Fig. 9).

**Fig. 10:**
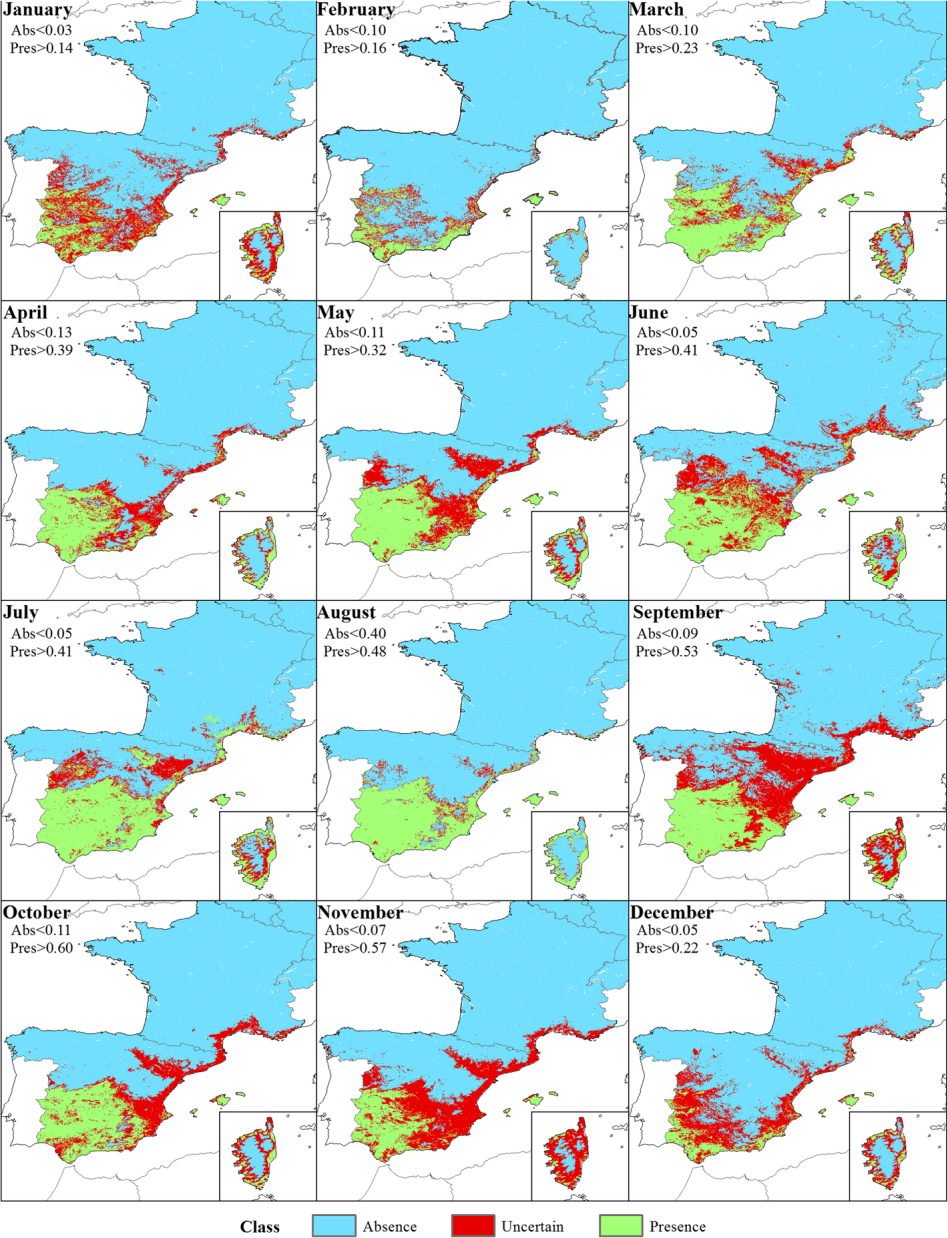
Classification of the predicted probability of presence of *C. imicola* into Absence, Presence and Uncertain areas at a 1 km^2^ resolution

Compared to the models for the Obsoletus ensemble, the models for *C. imicola* resulted in a clearer geographical division into three separate coherent zones. *Culicoides imicola* was found to be present in January and February in some areas in southern Spain, the Balearic Islands and Corsica. Uncertain areas were identified in central Spain, while the Absence regions were located in northern Spain and most of France with the exception of the southern coast. From March onwards, the Presence region extended northwards, occupying the southern and central regions of Spain until October, when it retracted back to the southern coast of Spain during late autumn. On Corsica, the Presence areas were located around the coast, with the vector being absent inland. The Uncertain area was always clearly located between the Presence and Absence areas and was generally small due to the high accuracy of the model in distinguishing between Presence and Absence classes (Fig. 10).

### Important predictors

The most important predictors driving the distribution of the Obsoletus ensemble, Pulicaris ensemble and *C. imicola* were related to temperature and precipitation for most months (dLST_MN, nLST_A0, nLST_MX, BIO 10, BIO 18, BIO 5). EVI- and NDVI-derived variables were the most important for some months and for some of the taxa, but with lesser importance compared to temperature and precipitation. Corine land cover classes were not selected as important variables and only one class (CLC 12: non irrigated arable land) was selected for Pulicaris during August. A similar situation occurred for the animal density variables, in which the only variable appearing in the top 5 most important variables was sheep density for the Pulicaris ensemble. Altitude was selected as an important variable only for the Obsoletus and Pulicaris ensembles, for the month of December (Additional file 1).

## Discussion

This study was based on the most extensive *Culicoides* dataset created to date. For these prediction maps, we used 31,429 *Culicoides* trap catches from nine European countries from 2007 to 2013 [33]. The objectives of this work were to predict the monthly probability of *Culicoides* presence and to demarcate regions of Europe into three presence classes, each for *C. imicola* and the Obsoletus and Pulicaris ensembles. We also identified areas and periods when the model was not able to predict with reasonable certainty. In these areas, targeted entomological surveillance programs implemented by the CVO’s of European Union member states are needed to clarify the present entomological status in case of an outbreak. The maps presented here can be used to determine vector-free areas (Absence areas) and areas where the vector can be found. The Absence and Presence areas were delimitated to minimize misclassification errors, making these classes more accurate in terms of the occurrence of *Culicoides*.

The models generated for the Obsoletus ensemble performed well for all months, and we were able to detect a spatial pattern in the three classes. However, the Absence and Presence classes were not completely separated by the model, and some geographical areas with Uncertain status were found among the Presence or Absence areas. For some of the months, our RF models were not able to clearly distinguish the minority class from the majority class, resulting in the threshold from the gain function being moved to the extremes to avoid misclassifications. This, in turn, resulted in a large Uncertain area that should potentially be targeted for costly entomological surveillance. This was the case for the Obsoletus ensemble during August, when the vector was indeed present in most of Europe but where our models classified the status as Uncertain in many smaller areas. For instance, in December the model predicted a large Uncertain status area that occupied most of the Scandinavia peninsula while the cold winter conditions make it unlikely that specimens will be found in northern Scandinavia. The Uncertain status areas should be interpreted with care and expert knowledge must be considered when making decisions regarding implementation of surveillance programs. The maps presented here are merely intended as tools and inputs to decision makers for long-term planning and in case of outbreaks in areas without ongoing entomological surveillance. The presented maps are based on a given gain function, but the gain function should reflect the severity of the vector borne diseases with an increasing emphasis on sensitivity as the severity of a disease increases.

In our models, the most important variables for the Obsoletus ensemble were the minimum daytime land surface temperature in January and February, and temperature- and precipitation-related variables (BIO 5 and BIO 14) throughout the rest of the year. Our results are in agreement with the findings of Calvete et al. [55] and Ducheyne et al. [56] who stated that temperature-related variables were the most important for the Obsoletus group distribution in Spain. Additionally, Purse et al [57] found that temperature had an effect in the occurrence of *C. obsoletus* in Italy. The Obsoletus ensemble are Palaearctic species requiring relatively low temperatures and humid climates for optimal development and survival [58, 59]. Temperature plays an important role in *Culicoides* ecology as it determines the seasonal fluctuation of the vector populations [60, 61], while humidity has been reported to create the optimal conditions for *C. obsoletus* breeding sites (e.g. dung heaps) [62].

To date, maps showing the PP and distribution of the Obsoletus ensemble for the entire Europe are scarce and incomplete. EFSA developed a website displaying distribution maps of *Culicoides* spp. On this site, a map of *C. obsoletus*/*C. scoticus* shows the distribution of this species [63] but the map is lacking information from some countries in Europe. At country level, some studies predicted the probability of Obsoletus group presence based on entomological data collected [56, 64–66]. Therefore, there is a need for predictions on a continental scale summarizing historical surveillance data to allow CVO’s of EU Member States to make rapid decisions in case of a future outbreak, as it would provide them with information on which areas and which time periods are likely to be vulnerable, which are likely to be safe and where the resources for surveillance should be allocated.

The RF models for the Pulicaris ensemble had poorer predictive power compared to Obsoletus ensemble and *C. imicola*. The abundance of the Pulicaris ensemble was ten-fold less than the abundance of the Obsoletus ensemble [33]. This lead to a lower number of Presence farms and, therefore, when the data were split into training and test sets, only a few Presence points were present in the test set. This resulted into heavily imbalanced monthly datasets e.g. February only included three farms with Presence observations in the test set. It is not recommended to assess model performance based only on a couple of observations from a certain class because it might lead to results with high variability. *Culicoides pulicaris* (*sensu stricto*) has been implicated in BTV transmission [67], but the Pulicaris ensemble species is not thought to have played a significant role in the 2006 BT outbreak in northern Europe [16]. Nevertheless, species of this ensemble might play a role in future outbreaks of emerging infections.

The model performance for *C. imicola* was highly accurate, with high AUC values for all months, indicating that this species has particular environmental requirements that can be detected through satellite imagery. This is likely to be related to hot and dry summers with low seasonal variation [64]: characteristic of the Mediterranean basin. The three classes were clearly distinguishable in the maps, and Presence and Uncertain areas could be delimited to the Iberian Peninsula. *Culicoides imicola* maps can be used directly to allocate resources for surveillance programs or to determine appropriate animal movement restrictions.

In our models, the most important explanatory variables for classification of areas for the Presence/Absence affecting *C. imicola* distribution were related to temperature and precipitation. We found that during winter, the mean temperature of the coldest quarter was the variable driving the presence of *C. imicola*, while variables related to precipitation were the most predominant drivers during the warmer months. This is in accordance with the results of previous studies [56, 64, 68].

The distribution of *C. imicola* has previously been modelled at continental level using classical statistical models fitted to data collected from single European countries [57, 64, 69]. In our maps, *C. imicola* appeared to be present all year round, as it can be found on the southern coast of Spain during January and February. This agrees with previous analyses of the start of the vector season in Europe, where *C. imicola* was found to be present during the winter months in southern Spain and central and southern Portugal [65]. The predicted probability of presence shown in our maps are in agreement to the distribution models made for Spain by Ducheyne et al. [56], Calvete et al. [55] and Peters et al. [70], and for France, where the Presence areas for the species are mainly located in the coastal regions of Corsica and VAR department [15].

In our study, we used *Culicoides* data aggregated into groups, namely the Obsoletus and Pulicaris ensembles. Aggregating species into a single group, or ensemble, might represent a challenge for ecological modelling, as the different species might require different environmental conditions and phenology differ between them. This has been studied by Searle et al. [24], who estimated the start and end date of the vector season and length of the vector-free period for four species of the Obsoletus ensemble. They observed that there were differences in phenology among the species. The lower model performance obtained for Obsoletus and Pulicaris ensembles compared to *C. imicola* may reflect that different species within each ensemble have different phenology and different environmental drivers. It would therefore be useful to identify *Culicoides* specimens to the species level. Molecular techniques, such as high-throughput real-time RT-PCR assays, can be used in a fast way for species identification. More accurate results could be expected if modelling is carried out on individual species data.

In practice, maps based on the classifications made for each 1 km^2^ pixel might be difficult to use for decision making, as it becomes challenging to define classes for larger areas in which pixels from different classes are found. For practical use, predicted pixel values may therefore be summarized by area, such as at NUTS level (nomenclature of territorial units for statistics) defined by Eurostat (2013). This would facilitate the implementation of control and surveillance programs by European veterinary authorities.

Random Forest is a machine learning technique that has previously been used for ecological species modelling [19, 56, 70–75]. This technique has been proven to perform better compared to other applications of classical statistical methods such as Non-Linear Discriminant Analysis and Generalized Ginear Models [19, 71], as well as Linear Discriminant Analysis, logistic regression [70, 74] and Additive Logistic Regression [75]. In this work, the monthly predicted probability of *Culicoides* presence had medium-high accuracy, but it is important to keep in mind that there might be other variables that cannot be captured by satellite imagery and that may have an influence on the occurrence of these species on a local scale, such as soil conditions (affecting breeding sites) and farming practices. Nevertheless, for some months, our models performed slightly better than other RF models used for predicting the occurrence of biting midges and mosquitoes [70, 71]. This highlights the challenges faced in predicting the occurrence of insect vectors using remote sensing data, as vectors are highly influenced by local microenvironments [76] and these data are difficult to obtain from satellite images without high spatial resolution.

## Conclusions

We present here maps as a risk assessment tool that can be used in the future to predict potential risk areas and risk seasons for *Culicoides*-borne disease outbreaks. They are particularly useful for European veterinary authorities, who can classify both areas likely to have vectors and likely to be vector-free in advance and during a sudden outbreak in areas without active entomological surveillance. Predicting areas of uncertain status allows focusing costly active entomological surveillance to limited areas. The developed gain functions used to delimit the areas for targeted active surveillance can easily be adjusted to new diseases where the cost of concluding false presence or false absence may be different than suggested here.

## Additional file

Additional file 1: **Table S1.** The top five of the most important variables by species group for each month. The variable importance is scaled from 0 to 100. Within each month (columns), the most important variable has a value of 100. (XLSX 67 kb)

## Abbreviations

Abs: Absence class
AUC: Area under the ROC curve
BT: Bluetongue disease
BTV: Bluetongue virus
CLC: Corine Land Cover
CV: Cross-validation
CVO: Chief Veterinary Officer
dLST: Daytime land surface temperature
MIR: Mid-infrared
NDVI: Normalized difference vegetation index
nLST: Nighttime land surface temperature
NUTS: Nomenclature of territorial units for statistics
PP: Probability of presence
Pres: Presence class
RF: Random Forest
ROC: Receiver operating characteristics curve
SVFP: Seasonal vector-free period
TFA: Temporal Fourier analysis

## References

[1] Du Toit RM (1944). The transmission of blue-tongue and horse-sickness by *Culicoides*. Onderstepoort J Vet Sci Anim Ind..

[2] Elbers ARW, Meiswinkel R, van Weezep E (2013). Sloet van Oldruitenborgh-Oosterbaan MM, Kooi EA. Schmallenberg virus in *Culicoides* spp. biting midges, the Netherlands, 2011. Emerg Infect Dis..

[3] Mellor PS, Boned J, Hamblin C, Graham S (1990). Isolations of African horse sickness virus from vector insects made during the 1988 epizootic in Spain. Epidemiol Infect..

[4] Mellor PS, Carpenter S, Harrup L, Baylis M, Mertens PPC (2008). Bluetongue in Europe and the Mediterranean Basin: history of occurrence prior to 2006. Prev Vet Med..

[5] Carpenter S, Wilson A, Mellor PS (2009). *Culicoides* and the emergence of bluetongue virus in northern Europe. Trends Microbiol..

[6] Toussaint J-F, Sailleau C, Mast J, Houdart P, Czaplicki G, Demeestere L (2007). Bluetongue in Belgium, 2006. Emerg Infect Dis..

[7] Thiry E, Saegerman C, Guyot H, Kirten P, Losson B, Rollin F (2006). Bluetongue in northern Europe. Vet Rec..

[8] Mehlhorn H, Walldorf V, Klimpel S, Jahn B, Jaeger F, Eschweiler J (2007). First occurrence of *Culicoides obsoletus*-transmitted bluetongue virus epidemic in central Europe. Parasitol Res..

[9] Zientara S, Sánchez-Vizcaíno JM (2013). Control of bluetongue in Europe. Vet Microbiol..

[10] Pinior B, Brugger K, Kofer J, Schwermer H, Stockreiter S, Loitsch A (2015). Economic comparison of the monitoring programmes for bluetongue vectors in Austria and Switzerland. Vet Rec..

[11] Rushton J, Lyons N (2015). Economic impact of bluetongue: a review of the effects on production. Vet Ital..

[12] Hoffmann B, Bauer B, Bauer C, Bätza HJ, Beer M, Clausen PH (2009). Monitoring of putative vectors of bluetongue virus serotype 8, Germany. Emerg Infect Dis..

[13] Carpenter S, Mcarthur C, Selby R, Ward R, Nolan DV, Mordue Luntz AJ (2008). Experimental infection studies of UK *Culicoides* species midges with bluetongue virus serotypes 8 and 9. Vet Rec..

[14] Dijkstra E, van der Ven IJK, Meiswinkel R, Holzel DR, van Rijn PA, Meiswinkel R (2008). *Culicoides chiopterus* as a potential vector of bluetongue virus in Europe. Vet Rec..

[15] Venail R, Balenghien T, Guis H, Tran A, Setier-Rio M-L, Delécolle J-C, Mehlhorn H (2012). Assessing diversity and abundance of vector populations at a national scale: example of *Culicoides* surveillance in France after bluetongue virus emergence. Arthropods as Vectors. Arthropods as Vectors of Emerging Diseases.

[16] Meiswinkel R, Baldet T, de Deken R, Takken W, Delécolle J-C, Mellor PS (2008). The 2006 outbreak of bluetongue in northern Europe - the entomological perspective. Prev Vet Med..

[17] Hartemink N, Vanwambeke SO, Purse BV, Gilbert M, Van Dyck H (2015). Towards a resource-based habitat approach for spatial modelling of vector-borne disease risks. Biol Rev..

[18] Hay SI, Tatem AJ, Graham AJ, Goetz SJ, Rogers DJ (2006). Global environmental data for mapping infectious disease distribution. Adv Parasitol..

[19] Cianci D, Hartemink N, Ibáñez-Justicia A (2015). Modelling the potential spatial distribution of mosquito species using three different techniques. Int J Health Geogr..

[20] Kalluri S, Gilruth P, Rogers D, Szczur M (2007). Surveillance of arthropod vector-borne infectious diseases using remote sensing techniques: a review. PLoS Pathog..

[21] EFSA Panel on Animal Health and Welfare. Bluetongue: control, surveillance and safe movement of animals. EFSA J. 2017;15:e04698. 10.2903/j.efsa.2017.4698 PMC7009973 32625424

[22] European Commission (2007). Ec 1266/2007. Off J Eur Union.

[23] Brugger K, Köfer J, Rubel F (2016). Outdoor and indoor monitoring of livestock-associated *Culicoides* spp. to assess vector-free periods and disease risks. BMC Vet Res..

[24] Searle KR, Barber J, Stubbins F, Labuschagne K, Carpenter S, Butler A (2014). Environmental drivers of *Culicoides* phenology: how important is species-specific variation when determining disease policy?. PLoS One..

[25] Kaufmann C, Steinmann IC, Hegglin D, Schaffner F, Mathis A (2012). Spatio-temporal occurrence of *Culicoides* biting midges in the climatic regions of Switzerland, along with large scale species identification by MALDI-TOF mass spectrometry. Parasit Vectors..

[26] Ander M, Meiswinkel R, Chirico J (2012). Seasonal dynamics of biting midges (Diptera: Ceratopogonidae: *Culicoides*), the potential vectors of bluetongue virus, in Sweden. Vet Parasitol..

[27] Mehlhorn H, Walldorf V, Klimpel S, Schmahl G, Al-Quraishy S, Walldorf U (2009). Entomological survey on vectors of bluetongue virus in Northrhine-Westfalia (Germany) during 2007 and 2008. Parasitol Res..

[28] Clausen P-H, Stephan A, Bartsch S, Jandowsky A, Hoffmann-Köhler P, Schein E (2009). Seasonal dynamics of biting midges (Diptera: Ceratopogonidae, *Culicoides* spp.) on dairy farms of central Germany during the 2007/2008 epidemic of bluetongue. Parasitol Res..

[29] Kiel E, Liebisch G, Focke R, Liebisch A (2009). Monitoring of *Culicoides* at 20 locations in northwest Germany. Parasitol Res..

[30] Afonso A, Abrahantes JC, Conraths F, Veldhuis A, Elbers A, Roberts H (2014). The Schmallenberg virus epidemic in Europe - 2011–2013. Prev Vet Med..

[31] Hoffmann B, Scheuch M, Höper D, Jungblut R, Holsteg M, Schirrmeier H (2012). Epizootic of ovine congenital malformations associated with Schmallenberg virus infection. Emerg Infect Dis..

[32] Ortega MD, Mellor PS, Rawlings P (1998). Pro MJ. The seasonal and geographical distribution of *Culicoides imicola*, *C. pulicaris* group and *C. obsoletus* group biting midges in central and southern Spain. Arch Virol Suppl..

[33] Cuéllar AC, Kjær LJ, Kirkeby C, Skovgard H, Nielsen SA, Stockmarr A (2018). Spatial and temporal variation in the abundance of *Culicoides* biting midges (Diptera: Ceratopogonidae) in nine European countries. Parasit Vectors..

[34] Nielsen SA, Nielsen BO, Chirico J (2010). Monitoring of biting midges (Diptera: Ceratopogonidae: *Culicoides* Latreille) on farms in Sweden during the emergence of the 2008 epidemic of bluetongue. Parasitol Res..

[35] Scharlemann JPW, Benz D, Hay SI, Purse BV, Tatem AJ, Wint GRW (2008). Global data for ecology and epidemiology: a novel algorithm for temporal fourier processing MODIS data. PLoS One..

[36] EDENext (2011). Biology and control of vector-borne infections in Europe.

[37] Hijmans RJ (2005). Worldclim - Global Climate Data. Free climate data for ecological modeling and GIS.

[38] Hijmans RJ, Cameron SE, Parra JL, Jones PG, Jarvis A (2005). Very high resolution interpolated climate surfaces for global land areas. Int J Climatol..

[39] European Environment Agency. Corine Land Cover. 2018. https://www.eea.europa.eu/data-and-maps/data/clc-2006-raster-4. Accessed 28 Oct 2018.

[40] Robinson TP, Wint GRW, Conchedda G, Van Boeckel TP, Ercoli V, Palamara E (2014). Mapping the global distribution of livestock. PLoS One..

[41] Random Forests BL (2001). Mach. Learn..

[42] Kuhn M, Johnson K (2013). Applied Predictive Modeling.

[43] R Core Team (2013). R: A language and environment for statistical computing.

[44] Kuhn M (2008). Building predictive models in R using the caret package. J Stat Softw..

[45] Liaw A, Wiener M (2002). Classification and regression by randomForest. R news..

[46] Breiman L (2001). Statistical modeling: The two cultures. Stat Sci..

[47] Guis H, Caminade C, Calvete C, Morse AP, Tran A, Baylis M (2012). Modelling the effects of past and future climate on the risk of bluetongue emergence in Europe. J R Soc Interface.

[48] Pearce J, Ferrier S (2000). Evaluating the predictive performance of habitat models developed using logistic regression. Ecol Modell..

[49] Lunardon N, Menardi G, Torelli NROSE (2014). A package for binary imbalanced learning. R J..

[50] Batista GEAPA, Prati RC (2004). Monard MC. A study of the behavior of several methods for balancing machine learning training data. ACM SIGKDD Explor Newsl..

[51] Fawcett T (2006). An introduction to ROC analysis. Pattern Recognit Lett..

[52] Guisan A, Zimmermann NE (2000). Predictive habitat distribution models in ecology. Ecol Modell..

[53] Mandrekar JN (2010). Receiver operating characteristic curve in diagnostic test assessment. J Thorac Oncol..

[54] Liu C, Berry PM, Dawson TP, Pearson RG (2005). Selecting thresholds of occurrence in the prediction of species distributions. Ecography..

[55] CALVETE C, ESTRADA R, MIRANDA M. A, BORRÁS D, CALVO J. H, LUCIENTES J (2008). Modelling the distributions and spatial coincidence of bluetongue vectors Culicoides imicola and the Culicoides obsoletus group throughout the Iberian peninsula. Medical and Veterinary Entomology.

[56] Ducheyne E, Miranda Chueca MA, Lucientes J, Calvete C, Estrada R, Boender G (2013). Abundance modelling of invasive and indigenous *Culicoides* species in Spain. Geospat Health.

[57] Purse BV, Tatem AJ, Caracappa S, Rogers DJ, Mellor PS, Baylis M (2004). Modelling the distributions of *Culicoides* bluetongue virus vectors in Sicily in relation to satellite-derived climate variables. Med Vet Entomol..

[58] Purse BV, Brown HE, Harrup L, Mertens PPC, Rogers DJ (2008). Invasion of bluetongue and other orbivirus infections into Europe: the role of biological and climatic processes. Rev Sci Tech..

[59] Brugger K, Rubel F (2013). Characterizing the species composition of European *Culicoides* vectors by means of the Köppen-Geiger climate classification. Parasit Vectors..

[60] Purse BV, Carpenter S, Venter GJ, Bellis G, Mullens BA (2015). Bionomics of temperate and tropical *Culicoides* midges: knowledge gaps and consequences for transmission of *Culicoides*-borne viruses. Annu Rev Entomol..

[61] Lühken R, Steinke S, Hoppe N, Kiel E (2015). Effects of temperature and photoperiod on the development of overwintering immature *Culicoides chiopterus* and *C. dewulfi*. Vet Parasitol..

[62] Steinke S, Lühken R, Balczun C, Kiel E (2016). Emergence of *Culicoides obsoletus* group species from farm-associated habitats in Germany. Med Vet Entomol..

[63] EFSA (2017). A story map. Bluetongue virus (BTV).

[64] Purse B, Mccormick BJJ, Mellor PS, Baylis M, Boorman JPT, Borras D (2007). Incriminating bluetongue virus vectors with climate envelope models. J Appl Ecol..

[65] Ramilo DW, Nunes T, Madeira S, Boinas F, da Fonseca IP (2017). Geographical distribution of *Culicoides* (Diptera: Ceratopogonidae) in mainland Portugal: presence/absence modelling of vector and potential vector species. PLoS One..

[66] Calvete C, Estrada R, Miranda MA, Borrás D, Calvo JH, Lucientes J (2009). Ecological correlates of bluetongue virus in Spain: predicted spatial occurrence and its relationship with the observed abundance of the potential *Culicoides* spp. vector. Vet J..

[67] Caracappa S, Torina A, Guercio A, Vitale F, Calabrò A, Purpari G (2003). Identification of a novel bluetongue virus vector species of *Culicoides* in Sicily. Vet Rec..

[68] Wittmann EJ, Mellor PS, Baylis M (2001). Using climate data to map the potential distribution of *Culicoides imicola* (Diptera: Ceratopogonidae) in Europe. Rev Sci Tech..

[69] Tatem AJ, Baylis M, Mellor PS, Purse BV, Capela R, Pena I (2003). Prediction of bluetongue vector distribution in Europe and north Africa using satellite imagery. Vet Microbiol..

[70] Peters J, De Baets B, Van Doninck J, Calvete C, Lucientes J, De Clercq EM (2011). Absence reduction in entomological surveillance data to improve niche-based distribution models for *Culicoides imicola*. Prev Vet Med..

[71] Ibañez-Justicia A, Cianci D (2015). Modelling the spatial distribution of the nuisance mosquito species *Anopheles plumbeus* (Diptera: Culicidae) in the Netherlands. Parasit Vectors..

[72] Ducheyne E, Charlier J, Vercruysse J, Rinaldi L, Biggeri A, Demeler J (2015). Modelling the spatial distribution of *Fasciola hepatica* in dairy cattle in Europe. Geospat Health..

[73] Selemetas N, Ducheyne E, Phelan P, O’Kiely P, Hendrickx G, de Waal T (2015). Spatial analysis and risk mapping of *Fasciola hepatica* infection in dairy herds in Ireland. Geospat Health..

[74] van Doninck J, De Baets B, Peters J, Hendrickx G, Ducheyne E, Verhoest NEC. Modelling the spatial distribution of *Culicoides imicola*: climatic versus remote sensing data. Remote Sens. 2014;6:6604–19.

[75] Cutler DR, Edwards TC, Beard KH, Cutler A, Hess KT, Gibson J (2007). Random Forests for classification in ecology. Ecology..

[76] Haider N, Cuellar AC, Kjær LJ, Sørensen JH, Bødker R (2018). Microclimatic temperatures at Danish cattle farms, 2000–2016: quantifying the temporal and spatial variation in the transmission potential of Schmallenberg virus. Parasit Vectors..

